# Characteristics, management and factors associated with poor outcomes in COVID-19 patients in Burkina Faso: insights from a 2021 large-scale ambispective study

**DOI:** 10.3389/fpubh.2025.1542024

**Published:** 2025-07-10

**Authors:** Ariane Mamguem Kamga, Samiratou Ouédraogo, Firmin Nongodo Kaboré, Isidore Tiandiogo Traoré, Esperance Ouédraogo, Armel Poda, Arnaud Eric Diendéré, Dramane Kania, Hermann Badolo, Guillaume Sanou, Amariane Koné, Therese Samdapawindé Kagoné, Blahima Konaté, Rachel Médah, Nathalie de Rekeneire, Boukary Ouédraogo, Oumar Billa, Gilles Paradis, Halidou Tinto, Tienhan Sandrine Dabakuyo-yonli

**Affiliations:** ^1^Centre Georges François Leclerc, Dijon, France; ^2^National Population Health Observatory, National Institute of Public Health, Ouagadougou, Burkina Faso; ^3^MURAZ Center, Bobo-Dioulasso, Burkina Faso; ^4^Centre National de la Recherche Scientifique et Technologique, Ouagadougou, Burkina Faso; ^5^Department of Infectious Diseases, Sourô Sanou University Hospital, Bobo Dioulasso, Burkina Faso; ^6^Higher Institute of Health Sciences, Nazi Boni University, Bobo Dioulasso, Burkina Faso; ^7^Bogodogo University Hospital, Ouagadougou, Burkina Faso; ^8^Expertise France, Paris, France; ^9^McGill University, Montreal, QA, Canada; ^10^Nanoro Clinical Research Unit, Health Sciences Research Institute, National Centre for Scientific and Technological Research, Bobo Dioulasso, Burkina Faso

**Keywords:** age, comorbidity, COVID-19, complications, treatment

## Abstract

**Objectives:**

To assess treatment and identify predictive factors of worsening in COVID-19 patients.

**Methods:**

This study was ambispective (both prospective and retrospective) and part of a multidisciplinary, multicenter project designed to generate epidemiological, sociological and anthropological data about the COVID-19 epidemic in Burkina Faso. Medical records of patients admitted for COVID-19 at the hospitals of Ouagadougou and Bobo-Dioulasso from March 2020 to April 2021 were reviewed. To identify predictive factors of severe complications, we used Poisson regression models.

**Results:**

In total, 1,511 patients were included, of whom 70% were aged ≤50 years, 59% were men and 97% were living in an urban area. Of the 86% of patients treated, 92.9% of them received the combo Azithromycin-hydroxychloroquine. A total of 78 (5.2%) patients experienced complications during hospitalization, and 49 (3.3%) patients died. Multivariate analysis identified patient's age, residence and comorbidity as factors associated with poor outcomes.

**Conclusions:**

Although most people had symptoms, most of them recovered without sequelae, and few patients had severe forms of disease. Age was a strong predictor of worse outcomes in this population.

## Introduction

Coronavirus diseases 19 (COVID-19) remains a global pandemic that continues to strain healthcare systems. Although most often benign, it is estimated that around 14% of infected individuals will develop a severe form of infection (1).

Northern countries were the most severely affected by COVID-19 at the beginning of the pandemic, and given that their strong health systems struggled with the disease, the worst was predicted for Africa and other developing countries (2–4). As time passed by, the scenario did not evolve as predicted in Africa, raising several questions about population-acquired immunity, asymptomatic cases, the number of reported cases, whether or not PCR testing was routine (5–7), what treatment to administrate and what factors might predict worsening of the disease.

Burkina Faso is one of the countries affected by the COVID-19 in Africa (8), with the first cases diagnosed in March 2020. As of April 10, 2025, there have been 22,106 reported cases in the country (9), with Ouagadougou and Bobo-Dioulasso being the two most affected major cities (10). The response plan put in place by the government recommended that in addition to barrier measures to limit the spread of the virus, suspected cases of patients with COVID-19 should be identified and treated in health facilities requisitioned by the government in the acute phase the pandemic.

There is a growing body of literature examining the prognostic factors of COVID-19. Many factors have been identified, such as advanced age, sex, comorbidities, oxygen saturation, clinical signs and biological factors (11–14). However, patient and disease factors vary from region to region (15, 16).

Since the notification of the first cases in the country, several studies have been conducted in Burkina Faso to identify the best therapy to manage cases, or socio-anthropological and prognostic studies (10, 17–19). These studies were either conducted in the capital, Ouagadougou only (10, 17, 18), with data collection limited to a few health facilities and using mortality as the outcome criterion (18), or they focused on only one of the severe forms of COVID-19 (18). Moreover, there are few data on how COVID-19 patients were treated in Burkina-Faso.

Thus, the objective of this study was to assess how patients with COVID-19 infection managed in the hospitals of Ouagadougou and Bobo-Dioulasso, Burkina Faso were treated and identify factors associated with worsening and death.

## Methods

### Study design

This study was part of a large, multidisciplinary, multicenter project (20) designed to generate epidemiological, sociological and anthropological data about the COVID-19 epidemic in Burkina Faso. The present study is an ambispective study (i.e., both prospective and retrospective), and includes all cases admitted to the official COVID-19 management centers in Ouagadougou and Bobo Dioulasso, the two main cities of Burkina Faso, from March 2020 to April 2021.

### Inclusion and exclusion criteria

To be eligible, patients had to be hospitalized or have been hospitalized and managed for COVID-19 in the four referral hospitals in Ouagadougou and Bobo-Dioulasso: Souro-Sanou, Tengandogo, Bogodogo and Yagaldo Ouedraogo during the period of March 2020 to April 2021.

### Statement of ethical approval

This study is a part of a multicenter project that has been approved by the Ministry of Health (N° 2020-00952/MS/CAB/INSP/CM) and the National Ethics Committee for health research of Burkina Faso (N° 2020-8-140). This study was performed in accordance with the ethical standards of the national research committee, the principles of Good Clinical Practice and the 1964 Helsinki declaration and its later amendments. All study participant of this study has provided signed consent.

### Procedure

The prospective part of the study took place from September 13, 2020 to April 30, 2021. Medical records of patients admitted for COVID-19 in the hospitals of Ouagadougou and Bobo-Dioulasso were review. Data were collected using a questionnaire (see supplementary material). Patients were follow-up till death or discharge from the hospital.

### Outcome

The main outcome of this study was the proportion of severe complications. Severe complications were defined as the occurrence any one of the following: severe pneumonitis, acute respiratory distress syndrome (ARDS), sepsis, septic shock, coma or death in patients infected with SARS-CoV-2. The proportion of deaths was also assessed.

### Independent variables

The following variables were collected for all patients: age, sex, place of residence (urban vs. rural), clinical signs on admission and during hospitalization, comorbidities at admission, lifestyle habits (alcohol, smoking status) and context of contamination as self-reported by patients. Age was categorized into <50 years vs. 50 years or more.

Comorbidities were divided into two categories (No comorbidities vs. at least one comorbidity). Comorbidities included diabetes, high blood pressure (HBP), HIV and other chronic conditions (cardiovascular, neurological, respiratory, renal, rheumatological, hepatic disease). Oxygen saturation in room air at admission (SaO_2_) was dichotomized as SaO_2_ < 94% and SaO_2_ ≥ 94%.

A threshold of 15 was used to dichotomize the Glasgow score. Subjects were considered symptomatic if they had at least one of the symptoms listed in the case report form. Clinical signs on admission were grouped into the following symptom classes: general (fatigue, fever, chills, anorexia, multiorgan failure), respiratory (cough, dyspnea, wheezing, chest pain), neurological (disturbed consciousness, convulsions, headache, ageusia, anosmia), digestive (abdominal pain, diarrhea, nausea/vomiting), musculoskeletal (joint pain, myalgia), Ear Nose and Throat (sore throat, epistaxis, rhinitis) and other symptoms (rash, conjunctivitis, etc.).

### Statistical analyses

Categorical variables are presented as number and percentage, and continuous variables as mean ± standard deviation (SD), or median and range, as appropriate.

To identify predictive factors of severe complications, we constructed two Poisson regression models (21). The first was built to identify correlates of admission with severe complications. The second was to identify factors associated with the occurrence of severe complications (including death). For this latter model, we excluded patients who were admitted directly with severe complications.

We first performed univariable analysis. Eligible variables for the multivariable model were all those with *p*-value < 0.10 by univariable analysis. We then performed backward stepwise selection to obtain the final models. Finally, prognostic factors of time to death were determined using a Cox regression model. Results are reported as relative risk (RR) for Poisson regression models or hazard ratio (HR) for the Cox model with their 95% confidence intervals and *p*-values.

Test results were considered significant for *p*-values < 0.05 for descriptive analyses. Considering the multiple analyses we conducted, Bonferroni's correction was applied setting the *p*-value for the Poisson models to 0.017. All statistical analyses were performed using SAS version 9.4 (SAS Institute Inc., Cary, NC).

## Results

### Sociodemographic characteristics

In total, 1,511 people were hospitalized and managed for COVID-19 in the 4 referral teaching hospitals in Ouagadougou and Bobo-Dioulasso. Among them, nearly 60% were living in Ouagadougou. The majority were men (59%), aged 50 years old or less (70%) and living in an urban area (98%; Table 1).

**Table 1:** Description of baseline characteristics of patients.

**Characteristics**	***N***	**%**
City		
Ouagadougou	902	59.7
Bobo-Dioulasso	609	40.3
Sex		
Male	896	59.3
Female	615	40.7
Residence		
Urban	1,478	97.9
Rural	32	2.1
Unknown	1	
Age		
< 50 years	1,057	70.0
≥50 years	454	30.0
Age (y)		
Mean (SD)	40.9 (15.9)	
Median [Min–Max]	39 [0.2–101]	
Comorbidities (yes)	446	30.3
High blood pressure	233	15.6
Diabetes	103	6.9
COPD/Asthma	60	4.0
Chronic kidney disease	41	2.7
Other cardiovascular disease	26	1.8
Chronic rheumatologic disease	18	1.2
Chronic hepatic disease	15	1.0
Chronic neurologic disease	11	0.7
HIV	6	0.4
Pulmonary tuberculosis	3	0.2
Malignant tumor	2	0.1
Others chronic disease	95	6.4
BCG vaccine	799	53.1
Alcohol (yes)	131	8.7
Smoking (No)	1,078	91.6
Clinical signs (yes)	1,198	79.3
Glasgow < 15	117	8.3
O2 saturation		
≥94%	1,239	92.2
< 94%	105	7.8
Unknown	167	

BCG, Bacille Calmette-Guerin; COPD, chronic obstructive pulmonary disease; HIV, human immunodeficiency virus; SD, standard deviation.

### Clinical features

The population was relatively in good health overall (87% in good general condition), with few comorbidities (30% had comorbidities) and a generally healthy lifestyle, with few smokers and consumers of alcohol. The most frequent comorbidities were diabetes (7%), HBP (16%) and chronic obstructive pulmonary disease (COPD)/asthma (4%; Table 1).

**Figure 1:**
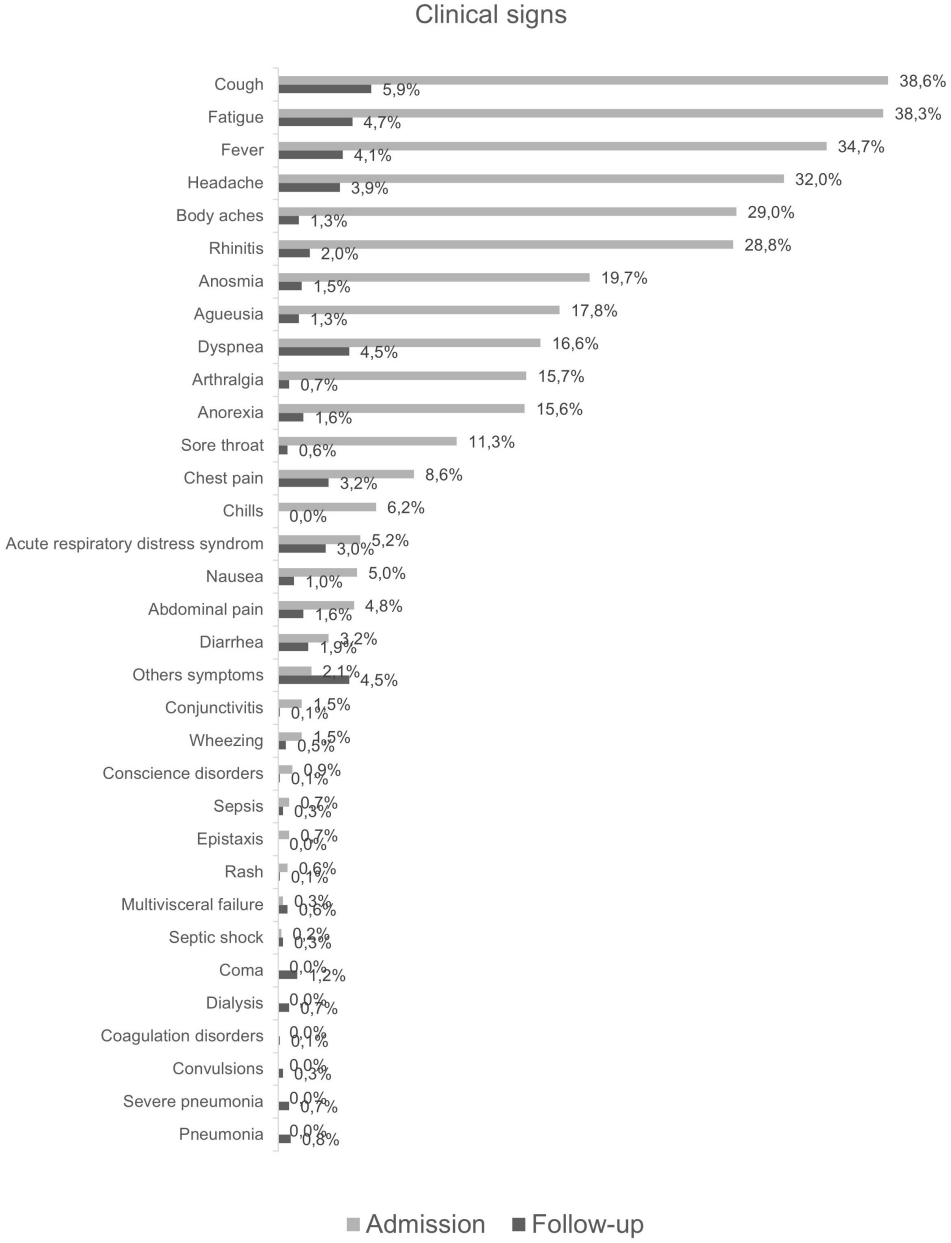
Description of symptoms at admission and during hospitalization.

**Figure 2:**
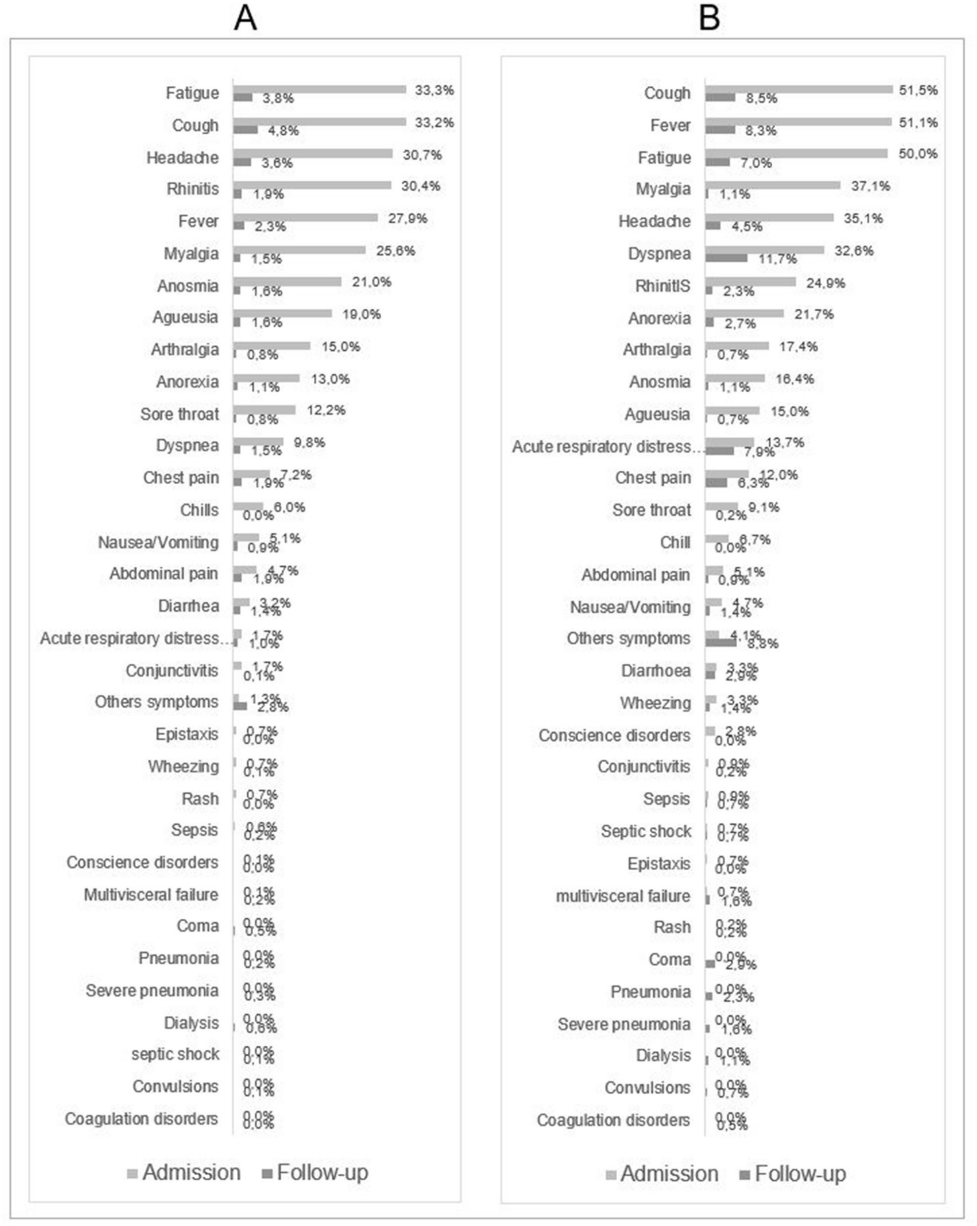
Description of symptoms at admission and during hospitalization for patients aged < 50 years **(A)** and patients aged 50 years or more **(B)**.

Nearly 4 out of 5 patients had symptoms on admission. The main symptoms were cough (38.6%), fatigue (38%), fever (35%), headache (32%), body aches (29%) and rhinitis (29%). Only 16% of patients had dyspnea (Figure 1). People aged 50 years and older had more symptoms compared to those aged < 50 years. Symptoms such as dyspnea, cough, fever and fatigue were more likely to persist at discharge in older patients (Figure 2).

Among younger patients, the most persistent symptom at discharge was cough (?5%) (Figure 2). Four percent of patients had auto medication before attending medical facilities.

### Treatment and outcomes

Eighty-six percent of patients received treatment. Among them, 92.9 % had been treated by the combo Azithromycin and hydroxychloroquine, 11.4% had received antibiotherapy other than azithromycin and 12% had symptomatic treatments (paracetamol, etc.). One hundred and thirty-four (9.4%) patients had oxygen therapy and 77 (5.4%) patients had tracheal intubation.

In our study population, 82 (5.7%) patients arrived at the hospital with severe forms, 78 (5.2%) patients had complications during hospitalization, 49 (3.3%) patients died. There was a difference in terms of complications and death according to age (*p* = 0.001) but no difference was found when comparing men to women (*p* = 0.001). Overall, 90% of patients recovered without sequelae (Table 2).

**Table 2:** Description of outcomes and treatments.

**Variables**	***N***	**%**
Complications during follow-up (yes)	78	5.2
Death		
Yes	49	3.3
No	1,443	96.7
Mode of discharge		
Cured without sequelae	1,239	92.2
Cured with sequelae	4	0.3
Discharge again medical advice	9	0.6
Referred	68	4.6
Out of isolation and not cured	17	1.1
Dead	49	3.3
Treatment (yes)	1,310	88.2

### Correlate and predictive factors

Age and residence were both predictors of admission with complications (age ≥ 50yrs; RR [95% CI]: 4.71 [2.56 – 8.66]; rural residence, RR [95% CI]: 2.67 [1.37 – 5.21]) and were also predictive factors associated with the occurrence of complications during hospitalization (age ≥ 50yrs RR [95% CI]: 3.33 [1.24 – 8.98]; rural residence RR [95% CI]: 9.02 [2.34 – 34.79]) (Table 3).

**Table 3:** Predictive factors of severe form of disease at admission and complications during hospitalization.

**Variables**	**Severe form at admission**		***P*-value**	**Complications during hospitalization**		***P*-value**
	**RR**	**95%CI**		**RR**	**95%CI**	
Age			< 0.0001			0.015
< 50 years	Ref		Ref		
≥50 years	4.71	[2.56–8.66]		3.33	[1.24–8.98]	
Residence			0.0037			0.0014
Urban area	ref		ref		
Rural area	2.67	[1.37–5.21]		9.02	[2.34–34.79]	
Comorbidities		0.0139			
No	ref		–	–	
Yes	2	[1.15–3.48]		–	–	

Furthermore, comorbidities (RR [95% CI]: 2 [1.15 – 3.48]) were associated with an increased risk of admission with complications (Table 3). Only age (HR [95% CI]:11.81 [3.42 – 40.75]) and the presence of respiratory symptoms at admission (HR [95% CI]: 21.7 [2.75 – 172.08]) were prognostic factors of mortality (Table 3).

## Discussion

The aim of this study was to assess the treatment and identify the factors associated with complications (worsening and death) among patients admitted with COVID-19 infection to hospitals in the two major cities of Burkina Faso, namely Ouagadougou and Bobo-Dioulasso.

We found that among people with COVID-19 infection in Burkina-Faso, few (6%) were admitted to hospitals with severe forms, and only 5% had a complication during hospitalization. This result shows that patients managed for COVID-19 in the major cities of Burkina Faso mainly had a mild form of disease. One explanation may be that in our study, the population was young, with a median age of 39 years, and had a relatively healthy lifestyle, with few smokers or alcohol consumers.

The young age of our population may be a protective factor against the development of severe forms of SARS-CoV-2 infection. However, based on the current state of knowledge, people with comorbidities like diabetes or high blood pressure may develop severe forms of COVID-19. In our study, only 16% and 7% had high blood pressure and diabetes respectively.

These results are in line with the findings of other studies in Burkina Faso (10, 17, 18) and Africa (18, 22), reporting mild or moderate forms as the most commonly encountered forms in COVID-19 patients. Nevertheless, data about lifestyle habits were self-reported, and we cannot exclude the possibility that they may be underreported.

The combo azithromycin-hydroxychloroquine (AZ-HCQ) was the main treatment received by COVID-19 patients. At the first breakthrough of COVID-19, researchers around the globe have tested several drugs and AZ-HCQ was one of them. Later through the spread of the COVID-19 disease, its efficacy had become of topic for debate among peers and in the scientific community.

In Burkina Faso, an observational and prospective study (CHLORAZ) (23) set up in order to assess the safety of AZ-HCQ, had found AZ-HCQ to be well tolerated by COVID-19 patients. Later on, others studies (19, 24) had focused on efficacy of AZ-HCQ and had shown no harmful outcomes in COVID-19 patients who took AZ-HCQ.

Although 79% of patients in this study presented symptoms at admission for COVID-19, most (90%) recovered fully without sequelae at discharge. This again suggests that people in Burkina Faso admitted for COVID-19 predominantly had a benign form of disease. This is also support by the low level of intensive care use seen among the patients, with only 8% of participants in this study requiring oxygen therapy. Nevertheless, some symptoms still persist at the time of discharge, particularly in people older than 50 years old.

Concerning factors associated with complications, age was associated with both severe form of disease at admission, and worsening of the disease. People aged 50 years and older had a three- and four-fold increase risk of having a severe form or worsening of the disease respectively, compared to those aged under 50.

Since the pandemic began, age has been clearly identified as a strong factor for severe forms or developing complications in patients with COVID-19 (11, 12). In most studies, people aged 60 years or older are at increased risk of complications due to COVID-19 (6, 12, 16, 25). Our study shows that even before 60 years old, there is already a risk of developing complications due to COVID-19. Age was also significantly associated with the risk of death.

The area of residence was found to be associated with both a severe form, and worsening of the disease, whereby living in a rural area was significantly associated with the likelihood of having a severe form of disease at admission. Health facilities are often far from rural areas, making it difficult for people living in rural areas to have prompt access to healthcare facilities.

Aside from the distance to the healthcare facilities, a fear of out-of-pocket expenses, such as direct costs of treatment and indirect costs for transportation and accommodation, could be one reason why patients in rural areas do not attend health facilities. In addition, most people living in rural areas in Burkina Faso are farmers who live off their crops.

For these people, losing a day's work means a corresponding loss of income, so they only go to hospital as a last resort. This late presentation could at least partially explain the higher proportion of severe forms in those from rural areas. In our study, 16% of patients living in a rural area presented either acute respiratory distress syndrome or sepsis at admission, and people living in rural areas also had a higher risk of poor outcomes.

Comorbidities were significantly associated with patients having severe forms at admission. Diabetes and HBP were identified as being associated with worse outcomes in COVID-19 patients (19, 24). In our study, 30% of patients presented one or more comorbidities, and the most common were HBP (15.6%), diabetes (6.9%), and COPD/asthma (4%). Comorbidity was associated with the occurrence of complications during follow-up in patients with a benign form at admission in univariate analysis, but not in multivariate analysis.

We also looked at the effect of comorbidities individually, mainly high blood pressure, diabetes and COPD/asthma. Both HBP and diabetes were independently associated with worsening in COVID-19 patients by univariate analyses, but this association was not significant in multivariate analysis. These findings on HBP is in line with those of Ouedraogo et al. (20) who found that HBP was an independent factor for ARDS in univariate but not in multivariate analysis.

As for diabetes, our results are contrary to those found by Ouedraogo et al. (20) and Diendéré et al. (10), who respectively reported that diabetes was associated with ARDS and hypoxia in COVID-19 patients. This difference may be due to the time when the study was conducted. Indeed, Ouedraogo et al. (20) and Diendéré et al. (10) conducted their studies during the first wave, at a time when scientific knowledge about the disease was scarce.

Our study included both waves and after the first wave, diabetes was already established as risk factor for worsening of the disease. Thus, during the second wave, people with diabetes were known to be at risk of complications, and may have had more COVID-19 prevention protocols to reduce their risk of acquiring the infection. This is reflected by the proportion of COVID-19 patients with diabetes in these studies, namely 12.4% and 8.4% respectively for Diendéré et al. (10) and Ouedraogo et al. (20) and only 6.9% in our study.

Men were more frequently affected by COVID-19 in this study, with 59% of males in our population. However, gender was not significantly associated with complications or death, contrary to reports from other studies in Africa and around the world, where an association between sex and poor outcomes in COVID-19 patients has been identified (13, 26).

The strengths of this study include the data collection at four hospitals, which ensured a high number of patients and also reduced possible selection bias. Contrary to other studies conducted in Burkina Faso, our study included the city of Bobo-Dioulasso, which is one of the main cities, apart from Ouagadougou, most impacted by COVID-19. This study also has strengths, such as the use of the robust Poisson method (21) to identify predictive factors of worsening given, the low rate of complications in our study population.

However, this study also has some limitations. Although, we tested correlations between variables before including them in the models, we didn't calculate the variance inflation factor (VIF), which could have helped detect multicollinearity in the models and clarified the wide confidence intervals observed. Additionally, the exclusion of patients with complications at admission before performing the second regression model may introduce bias.

But, to mitigate this potential bias, we compared patients with complications at admission to those included in the model to assess whether there were significant differences between the two groups. The populations differed in terms of dyspnea, myalgia, abdominal pain and fatigue but only dyspnea (data not shown) was included in the model as part of the respiratory symptoms.

Furthermore, biological and radiological data were not included in the analysis of predictive factors. The reason be that these data were only available for a limited number of individuals at the hospitals where our data were collected. Burkina Faso is a low-income country where a large proportion of the population are working in the informal sector. Exams such laboratory tests or radiographs come with additional costs for patients, and given the socio-economic conditions, few individuals are able to afford them.

In conclusion, despite these limitations, the study offers valuable insights into the predictors of poor outcomes among COVID-19 patients in Burkina. While most patients had symptoms, the majority of patients in this young study population recovered without sequelae, and the overall proportion of complications was low. Patients were mainly treated with AZ-HCQ. The factors associated with poor outcomes are consistent with those found in the literature, including age, place of residence and comorbidities.

## Data Availability

The data that support the findings of this study are available from the corresponding author upon reasonable request.

## References

[1] Verity R Okell LC Dorigatti I Winskill P Whittaker C Imai N. Estimates of the severity of coronavirus disease 2019: a model-based analysis. Lancet Infect Dis. (2020) 20:669–77. 10.1016/S1473-3099(20)30243-7 32240634 PMC7158570

[2] El-Sadr WM Justman J. Africa in the path of COVID-19. N Engl J Med. (2020). 383:e11 10.1056/NEJMp2008193 32302075

[3] Cabore JW Karamagi HC Kipruto H Asamani JA Droti B Seydi ABW. The potential effects of widespread community transmission of SARS-CoV-2 infection in the World Health Organization African Region: a predictive model. BMJ Glob Health. (2020) 5:e002647. 10.1136/bmjgh-2020-002647 32451366 PMC7252960

[4] Sze S Pan D Nevill CR Gray LJ Martin CA Nazareth J. Ethnicity and clinical outcomes in COVID-19: a systematic review and meta-analysis. EClinicalMedicine. (2020) 29:100630. 10.1016/j.eclinm.2020.100630 33200120 PMC7658622

[5] Sagna T Ouedraogo P Traore L Obiri-Yeboah D Yonli A Tapsoba A. Enigma of the high prevalence of anti-SARS-CoV-2 antibodies in HIV-positive people with no symptoms of COVID-19 in Burkina Faso. J Public Health Afr. (2022) 13.1778. 10.4081/jphia.2022.1778 35720802 PMC9202456

[6] Kirenga BJ Byakika-Kibwika P. Excess COVID-19 mortality among critically ill patients in Africa. Lancet. (2021) 397:1860–1. 10.1016/S0140-6736(21)00576-6 34022973 PMC8137310

[7] Olumade TJ Uzairue LI. Clinical characteristics of 4499 COVID-19 patients in Africa: a meta-analysis. J Med Virol. (2021) 93:3055–61. 10.1002/jmv.26848 33543800 PMC8013423

[8] Coronavirus (COVID-19). WHO | Regional Office for Africa. Available online at: https://www.afro.who.int/health-topics/coronavirus-covid-19 (Accessed February 15, 2023).

[9] World Health Organization. Available online at: https://www.who.int/countries/bfa/ (Accessed April 10, 2025).

[10] Diendéré EA Sondo KA Ouédraogo AR Dahourou DL Cissé K Sawadogo A. Predictors of severe hypoxemia among COVID-19 patients in Burkina Faso (West Africa): findings from hospital based cross-sectional study. Int J Infect Dis. (2021) 108:289–95. 10.1016/j.ijid.2021.04.007 33894354 PMC8059284

[11] Sensusiati AD Amin M Nasronudin N Rosyid AN Ramadhan NA Lathifah R. Age, neutrophil lymphocyte ratio, and radiographic assessment of the quantity of lung edema (RALE) score to predict in-hospital mortality in COVID-19 patients: a retrospective study. F1000Res. (2021) 9:1286. 10.12688/f1000research.26723.2 33537125 PMC7836085

[12] Biswas M Rahaman S Biswas TK Haque Z Ibrahim B. Association of sex, age, and comorbidities with mortality in COVID-19 patients: a systematic review and meta-analysis. Intervirology. (2021) 64:36–47. 10.1159/000512592 33296901 PMC7801974

[13] Kragholm K Andersen MP Gerds TA Butt JH Østergaard L Polcwiartek C. Association between male sex and outcomes of coronavirus disease 2019 (COVID-19)-a Danish Nationwide, Register-based Study. Clin Infect Dis. (2021) 73:e4025-e4030. 10.1093/cid/ciaa924 32634827 PMC7454435

[14] Thoreau B Galland J Delrue M Neuwirth M Stepanian A Chauvin A. D-dimer level and neutrophils count as predictive and prognostic factors of pulmonary embolism in severe non-ICU COVID-19 patients. Viruses. (2021) 13:758. 10.3390/v13050758 33926038 PMC8146364

[15] Jang JG Hur J Choi EY Hong KS Lee W Ahn JH. Prognostic factors for severe coronavirus disease 2019 in Daegu, Korea. J Korean Med Sci. (2020) 35:e209. 10.3346/jkms.2020.35.e209 32537954 PMC7295599

[16] Nguimkeu P Tadadjeu S. Why is the number of COVID-19 cases lower than expected in Sub-Saharan Africa? A cross-sectional analysis of the role of demographic and geographic factors. World Dev. (2021) 138:105251. 10.1016/j.worlddev.2020.105251 33106726 PMC7577660

[17] Jaspard M Sow MS Juchet S Dienderé E Serra B Kojan R. Clinical presentation, outcomes and factors associated with mortality: a prospective study from three COVID-19 referral care centres in West Africa. Int J Infect Dis. (2021) 108:45–52. 10.1016/j.ijid.2021.05.024 34000419 PMC8120805

[18] Ouédraogo AR Bougma G Baguiya A Sawadogo A Kaboré PR Minougou CJ. Facteurs associés à la survenue de la détresse respiratoire aiguë et au décès chez des patients atteints de COVID-19 au Burkina Faso. Rev Mal Respir. (2021) 38:240–8. 10.1016/j.rmr.2021.02.001 33589360 PMC7862901

[19] Rouamba T Ouédraogo E Barry H Yaméogo NV Sondo A Boly R. Assessment of recovery time, worsening, and death among inpatients and outpatients with COVID-19, treated with hydroxychloroquine or chloroquine plus azithromycin combination in Burkina Faso. Int J Infect Dis. (2022) 118:224–9. 10.1016/j.ijid.2022.02.034 35227869 PMC8881228

[20] Traoré IT Ouedraogo S Kania D Kaboré FN Konaté B Médah R. COVID-19 epidemiological, sociological and anthropological investigation: study protocol for a multidisciplinary mixed methods research in Burkina Faso. BMC Infect Dis. (2021) 21:896. 10.1186/s12879-021-06543-4 34479501 PMC8414025

[21] Chen W Qian L Shi J Franklin M. Comparing performance between log-binomial and robust Poisson regression models for estimating risk ratios under model misspecification. BMC Med Res Methodol. (2018) 18:63. 10.1186/s12874-018-0519-5 29929477 PMC6013902

[22] Fouda Mbarga N Epee E Mbarga M Ouamba P Nanda H Nkengni A. Clinical profile and factors associated with COVID-19 in Yaounde, Cameroon: a prospective cohort study. PLoS ONE. (2021) 16:e0251504. 10.1371/journal.pone.0251504 33979402 PMC8115782

[23] Rouamba T Barry H Ouédraogo E Tahita MC Yaméogo NV Poda A. Safety of chloroquine or hydroxychloroquine plus azithromycin for the treatment of COVID-19 patients in Burkina Faso: an Observational Prospective Cohort Study. Ther Clin Risk Manag. (2021) 17:1187–98. 10.2147/TCRM.S330813 34815671 PMC8604637

[24] Baguiya A Poda A Cissé K Sondo AK Ouedraogo B et al. Effect of hydroxychloroquine or chloroquine and azithromycin on COVID-19 patients' recovery and mortality: evidence from a hospital based retrospective cohort study conducted in Burkina Faso. J Infect Dis Epidemiol. (2021) 7:192. 10.23937/2474-3658/1510192

[25] Sila T Suriyaamorn W Toh C Rajborirug S Surasombatpattana S Thongsuksai P et al. Factors associated with the worsening of COVID-19 symptoms among cohorts in community- or home-isolation care in southern Thailand. Front. Public Health, (2024) 12:1350304. 10.3389/fpubh.2024.1350304 38572011 PMC10987961

[26] Dalal J Triulzi I James A Nguimbis B Dri GG Venkatasubramanian A. COVID-19 mortality in women and men in sub-Saharan Africa: a cross-sectional study. BMJ Glob Health. (2021) 6:e007225. 10.1136/bmjgh-2021-007225 34815243 PMC8611236

